# Variability in the Incidence of miRNAs and Genes in Fragile Sites and the Role of Repeats and CpG Islands in the Distribution of Genetic Material

**DOI:** 10.1371/journal.pone.0011166

**Published:** 2010-06-17

**Authors:** Alessandro Laganà, Francesco Russo, Catarina Sismeiro, Rosalba Giugno, Alfredo Pulvirenti, Alfredo Ferro

**Affiliations:** 1 Department of Mathematics and Computer Science, University of Catania, Catania, Italy; 2 Department of Biomedical Sciences, University of Catania, Catania, Italy; 3 Imperial College Business School, Imperial College London, London, United Kingdom; Ohio State University Medical Center, United States of America

## Abstract

**Background:**

Chromosomal fragile sites are heritable specific loci especially prone to breakage. Some of them are associated with human genetic disorders and several studies have demonstrated their importance in genome instability in cancer. MicroRNAs (miRNAs) are small non-coding RNAs responsible of post-transcriptional gene regulation and their involvement in several diseases such as cancer has been widely demonstrated. The altered expression of miRNAs is sometimes due to chromosomal rearrangements and epigenetic events, thus it is essential to study miRNAs in the context of their genomic locations, in order to find significant correlations between their aberrant expression and the phenotype.

**Principal Findings:**

Here we use statistical models to study the incidence of human miRNA genes on fragile sites and their association with cancer-specific translocation breakpoints, repetitive elements, and CpG islands. Our results show that, on average, fragile sites are denser in miRNAs and also in protein coding genes. However, the distribution of miRNAs and protein coding genes in fragile versus non-fragile sites depends on chromosome. We find also a positive correlation between fragility and repeats, and between miRNAs and CpG islands.

**Conclusion:**

Our results show that the relationship between site fragility and miRNA density is far more complex than previously thought. For example, we find that protein coding genes seem to be following similar patterns as miRNAs, if considered their overall distribution. However, once we allow for differences at the chromosome level in our statistical analysis, we find that distribution of miRNA and protein coding genes in fragile sites is very different from that of miRNA. This is a novel result that we believe may help discover new potential correlations between the localization of miRNAs and their crucial role in biological processes and in the development of diseases.

## Introduction

Chromosomal fragile sites are heritable specific loci especially prone to breakage and rearrangements when cells are exposed to specific culture conditions or certain chemical agents such as inhibitors of DNA replication or repair [1], [2], [3]. They can be classified as rare or common, according to their frequency within the population. Rare fragile sites are present in a small fraction of the population and are usually associated with human genetic disorders, while common fragile sites are present in all individuals and, thus, represent a component of normal chromosome structure. A number of fragile sites span genes encoded by very large genomic regions. The observed rearrangements, affecting the associated genes, are usually insertions, deletions or translocations. Moreover, it has been shown that many genes involved in cancer-specific recurrent translocations are located within fragile sites [4]. This often results in the expression of altered oncogenes or the loss of tumor suppressors, contributing to the initiation of cancer [5], [6]. MicroRNAs [miRNAs] are endogenous small non coding RNAs responsible of post-transcriptional gene regulation [7]. They regulate specific target genes expression through the association with a large, multi-protein complex called RNA Induced Silencing Complex [RISC]. miRNAs into RISC recognize their targets by the binding of their bases to partially complementary sites usually located in the 3′ UTR region of their targets. However, functional miRNA binding sites can also occur within the 5′ UTR [8] or coding region [9]. miRNAs have been reported to be involved in many biological processes, including developmental timing, differentiation, proliferation, cell death, and metabolism [10], [11], [12]. Their oncogenicity has been demonstrated in a variety of cancers and their aberrant expression due to chromosomal rearrangements has been reported [13], [14]. For example, miR-15 and miR-16 are located at chromosome 13q14, a region deleted in B cell chronic lymphocytic leukemia [CLL], and it has been shown that both miRNAs are deleted or down-regulated in the majority of CLL cases [14], [15]. miRNA expression can also be regulated by epigenetic mechanisms [16]. Some miRNAs are down-regulated while others are over-expressed, and they can act as tumor-suppressor genes or oncogenes, respectively. Tumor-suppressor genes can be aberrantly methylated in cancer, and consequently down-regulated. The tumor-suppressor gene WWOX, located within the fragile site FRA16D, is correlated to multiple cancers, especially breast, prostate and ovary [17], [18]. A vastly studied mechanism of reducing WWOX at the transcriptional level is the hyper-methylation of CpG islands in its promoter and coding region [18]. Fragile sites are often characterized by repetitive sequences. Folate sensitive rare fragile sites have been found to represent loci with expansive mutations of the normally occurring CCG/CGG trinucleotide repeat sequences adjacent to a CpG island [19], [20], while non-folate sensitive rare fragile sites have been found to comprise polymorphic AT-rich minisatellite repeats [21], [22]. Fragile sites may also consist of other repetitive elements. For example, the nucleotide sequence of FRA6F is rich in repetitive elements like LINE1 and LINE2, Alu, MIR, MER and endogenous retroviral sequences and shows several DNA segments with increased helix flexibility [23]. Alu elements are the most abundant class of interspersed repeat sequences [24]. Recently, it was reported that some mammalian miRNAs are derived from genomic repeats and some of them show perfect complementarity with the MIR/LINE-2 class of repeat elements, which are present within a large number of human mRNAs and EST transcripts that contain portions of MIR and other LINE-2 elements in their 3′-untranslated regions [25]. It has been hypothesized that Alu elements within 3′-UTRs are targeted specifically by certain miRNAs [26]. Previous works showed that miRNA genes are frequently located at fragile sites and cancer-related genomic regions [6], [27], [28]. Here we present the complete mapping of the human miRNA genes on fragile sites, cancer-specific translocation breakpoints, repetitive sequences and CpG islands. The aim of this work is to highlight the potential connections between the localization of miRNAs and their role in biological processes and in the development of diseases.

## Results

### Overall fragile sites are particularly dense in miRNAs and protein coding genes

The mapping of human miRNA genes revealed that 242 of 715 miRNAs (33.8%) are located in chromosome fragile sites, sometimes overlapping with genes mapped on translocation breakpoints (see Table S1). This is a notable finding, considering that fragile sites account for about 25.8% of the length of all sites considered together and seems to indicate a higher than expected concentration of miRNA in fragile sites (see Table 1). In order to understand if this is a peculiarity of miRNAs we also mapped protein coding genes on fragile sites and obtained a similar percentage (7,446 of 21,945 genes, i.e. 33.9%, are located in fragile sites). Hence, it appears that the overall distribution of genetic material tends to be denser in fragile sites and that this is not only a characteristic of miRNA, unlike previous literature seemed to suggest. In addition, the analysis performed using a zero-inflated Poisson regression for the miRNAs and a Poisson regression model for the genes, reveals also that fragile sites contain more miRNAs and more genes than non-fragile regions (Fragile IRR_genes_ = 1.346; Fragile IRR_miRNAs_ = 1.523, conditioned on a non-zero value being observed; Fragile IRR_miRNAs_ = 1.354, unconditional, i.e., whether we observe a zero or a non-zero value). In this analysis, chromosome-specific effects are accounted for and the differing lengths of each region are considered as exposure controls (see Table 2). Though the results seem to indicate the tendency for a higher incidence of miRNAs in fragile sites when compared to genes (conditional and unconditional Fragile IRR_miRNAs_ > Fragile IRR_genes_), the difference is not statistically significant (p>0.05).

**Table 1:** Overall Descriptive Statistics.

	Fragile Regions	Non-Fragile Regions	Total
Number of regions	105	100	205
Average length (mbp)	7.45	22.42	14.76
miRNA			
Total number	242	473	715
Average per region	2.30	4.73	3.49
Average per unit of length	0.31	0.21	0.24
Protein coding genes			
Total number	7,446	14,499	21,945
Average per region	70.91	144.99	107.05
Average per unit of length	9.52	6.47	7.25

**Table 2:** Model Results — Fragile Incidence Rate Ratios (IRR).

	miRNA Model (conditional)	miRNA Model (unconditional)	Protein Coding Genes Model
Fragile IRR Estimate	1.523	1.366	1.347
95% Confidence Interval	[1.256, 1.845]	[1.151, 1.621]	[1.306, 1.388]

*Conditional IRR considers the impact of fragility given that the miRNA value in a region is greater than 0; unconditional IRR considers the impact of fragility overall, even when including zero-valued regions;

(ZIP model for miRNA and standard Poisson model for genes; model controls for differential exposure due to length and chromosome heterogeneity).

Moreover, 317 of 715 miRNAs (44.3%) are located within genes which are translocated in cancer and 87 of them (27.4%) are also in fragile sites. When looking in detail chromosome by chromosome, we find that chromosome 19 has the highest number of miRNAs and genes in fragile regions, while no miRNAs have been yet found in the fragile sites of chromosome 20 (Table S2). This result seems to suggest that incidence of genetic material might depend on the specific chromosome considered.

### The incidence of miRNAs on fragile versus non-fragile sites depends on chromosome

Though the previous analysis seems to demonstrate that there are little overall differences between miRNAs and protein coding genes with respect to their incidence across fragile versus non-fragile regions, further analysis reveals significant differences across chromosomes (see Table S3). miRNAs show a greater incidence in fragile regions within chromosomes 16, 19 and X (for example, Fragile IRR_miRNA_c19_ = 29.391, an extremely high value). However, within chromosome 14 we observe the opposite result: in this chromosome there is a lower incidence of miRNAs in fragile regions (Fragile IRR_miRNA_c14_ = 0.244). For all other chromosomes, fragile regions have neither a greater nor a lower incidence of miRNA. This means that the overall average result we reported previously was being driven by regions in few chromosomes (chromosomes 16, 19, and X). Indeed, once we allow for fragility to predict miRNA incidence differently in each chromosome (allow for the interaction of fragility dummy and chromosome) the overall effect of fragile (across all chromosomes) becomes insignificant (p>0.05). For genes we observe a more complex result. Chromosomes 2, 7, 8, 11, 12, 16, 19, 20, 22, and X show a greater incidence of genes in fragile regions. In contrast, chromosomes 5, 9, 13, and 17 show a lower incidence of genes in fragile regions. All other chromosomes do not show any significant incidence difference in fragile sites versus non-fragile regions (see Table S3). Note also that the extreme values we found in the incidence of miRNA were not detected for genes (the maximum Fragile IRR is IRR_genes_c19_ = 8.899). This is an important finding. Though chromosome 19 seems denser in fragile regions (higher incidence of both miRNAs and genes than remaining chromosomes), the incidence of miRNAs is 29 times higher versus 9 times higher for genes.

Hence, we conclude that the incidence of miRNAs in fragile sites is significantly different from that of protein coding genes when looking at specific chromosomes, though the overall incidence across all chromosomes seemed (after the first analysis) to be the similar.

### Sites with more repeats are slightly more likely to be fragile

The results of the Logit model analysis on site fragility, using repeats per unit of length and CpG islands per unit of length as predictors, show that fragile sites are positively correlated to repeats (coefficient_repeat_mpg = 0.002 with p = 0.026) but not to CpG islands (p>0.05). Conversely, when modeling repeats as a function of site fragility, chromosome dummies, miRNAs and gene count, we find that repeats are significantly and positively correlated to miRNAs and to the number of genes and that, on average, repeats are 10% more frequent on fragile sites. Interestingly, when we performed the same kind of analysis on CpGs, we found that on average CpGs are 32% more frequent on fragile sites though CpGs were not significant in the fragile Logit model (i.e., CpGs were not predictive of fragility but fragile sites tend to have a greater incidence of CpGs). CpGs are also positively correlated to miRNA and protein coding genes.

### Sites with more repeats are less likely to contain miRNA and genes; sites with more CpG islands are more likely to contain miRNA and genes

We re-estimated the miRNA and genes models introducing the repeats and CpG variables (in thousands, for better scaling). We find that miRNA and genes are less frequent with more repeats (Repeats IRR_miRNA_ = 0.985 and Repeats IRR_genes_ = 0.988, with p<0.05 for both) and that miRNA and genes are more frequent when there is a greater incidence of CpGs (CpG IRR_miRNA_ = 1.165 and CpG IRR_genes_ = 1.213, with p<0.05 for both). In addition, repeats and CpG results for miRNA and Genes do not present significant differences (the 95% confidence intervals in both models overlap and the test for their difference has p = 0.267). Table 3 summarizes these results.

**Table 3:** Repeat and CpG IRR Results for miRNA and Genes.

Variable	miRNA		Protein Coding Genes	
	IRR*	95% Confidence Interval	IRR	95% Confidence Interval
Repeats (in thousands)	0.985	[0.979, 0.992]	0.988	[0.978, 0.990]
CpG Islands (in thousands)	1.165	[1.067, 1.272]	1.213	[1.194, 1.232]

*Conditional and unconditional results are extremely similar in the case of Repeats and CpG islands; Here we will report only the conditional values; p<0.001 in all cases;

(Repeats and CPG variables are rescaled; we report on the ZIP model for miRNA and the Poisson model for genes; both models include chromosome dummies and the statistically significant interactions between chromosomes and site fragility; models also control for the differential exposure associated to differing site lengths).

### After accounting for the effects of Repeats and CpG islands we find that miRNA and Gene baseline incidence still varies significantly across chromosomes and the effect of site fragility (specific to each chromosome) is also still present

Even after accounting for the effects of Repeats and CpG islands on miRNA and gene incidence we observe that effects of site fragility specific to each chromosome are still statistically significant; and though some of the effects change slightly (e.g., updated Fragile IRR_miRNA_c19_ = 15.867 and updated Fragile IRR_genes_c19_ = 3.458) the overall results (direction and relative magnitude) hold (see Table S4). This means that the effect of site fragility seems not to be due solely to the stronger presence of repeats and CpG islands in those regions. There are other factors associated with site fragility beyond repeats and CpGs that are relevant for miRNA and gene incidence (because of the extremely high correlation between Repeats and CpGs at some of the chromosomes, we are unable to also include effects of repeats CpGs specific to each chromosome). Finally, also the baseline incidence of miRNA and genes in each chromosome vary significantly even after accounting for the effects of repeat and CpGs. This baseline incidence can be seen in Table S5 and should be further studied and linked to specific diseases (e.g., in chromosome X miRNAs are 3 times more frequent than average and protein coding genes are 23% less frequent than average).

## Discussion

Previous studies [6], [27] compared the genome positions of fragile sites and cancer susceptibility loci, with those of miRNAs in human and mouse. Results suggested a statistically significant association between the chromosomal locations of miRNAs and those of fragile sites and of regions involved in cancer. In our study we extended Calin's work [6] to *all* currently known human miRNAs (today there are more than 700 known miRNA, and at the time of Calin's work only about 200 were known). In addition, we also considered the location of protein coding genes and studied whether these followed similar patterns as miRNAs, an analysis that previous work had not considered. The results of our analysis show that fragile sites are particularly dense in miRNAs (confirming Calin's findings) *and* also in protein coding genes. Our overall initial results also indicated no significant difference between miRNAs and genes in terms of their distribution in fragile versus non-fragile sites.

We further extended the analysis to consider how the distribution of miRNAs and genes in fragile vs. non-fragile sites depended on chromosome. Our chromosome-specific results show that the distribution of miRNAs is actually significantly different from that of genes. For example, in chromosome 19 we observe that the incidence of miRNAs in fragile sites is twenty-nine times higher than in non-fragile regions, versus nine times higher for genes. Surprisingly we even find that, although on average regions with fragile sites are denser in miRNAs and genes, when looking at specific chromosomes sometimes the reverse happens. For example, in chromosome 14 there is a lower incidence of miRNAs in fragile regions. Hence, the incidence of miRNAs and of protein coding genes on fragile versus non-fragile sites depends on chromosome. This is a novel result that has not been presented in previous literature and that reveals that the distribution of miRNAs and genes is far more complex than previously thought.

Moreover, we also find that almost half of miRNAs are located near or within genes translocated in cancer.

Our data also show a positive correlation between chromosome fragility and repeats. mir-616 and mir-28 are derived from transposed elements (LINE, L2 family) [29] and are located within two translocation breakpoints, respectively DDIT3, that is often fused to FUS in myxoid liposarcoma [30], and LPP, that is fused to MLL in a secondary acute leukemia [31]. Moreover several human microRNAs are transcribed by RNA polymerase III through promoters and/or terminators derived from the Alu retrotransposon [32] and all these miRNA genes are located in chromosome 19 within FRA19A (5-azacytidine type, common). Chromosome 19 has a high Alu elements density, highly correlated with GC content [33]. A primate-specific gene cluster on chromosome 19 encodes the majority of miRNAs that show high seed complementarity to the most conserved sense Alu sites. A dual relationship exists between this evolutionary young miRNA cluster and their Alu targets that may have evolved in the same time window. One hypothesis for this dual relationship is that these miRNAs could protect against too high rates of duplicative transposition, which would destroy the genome [34].

The high correlation that we found between miRNAs and CpG island is consistent with some recent findings. Several miRNA loci, including miR-9-1, -193a, -137, -342, -203 and -34b/c, are found to be hypermethylated in multiple human cancers [35]. Conversely, the let-7a-3 locus was found to be hypomethylated in lung adenocarcinoma and elevated expression of this locus resulted in enhanced oncogenic gene transcription [16].

The expression of fragile sites could also be affected by the environment and other factors such as alcohol and smoke. mir-218 (miR-218-1) is down-regulated in smokers [36] and it is encoded within the intronic region of the known tumor suppressor gene SLIT2 located at 4p15.31. SLIT2, which is frequently inactivated in lung and breast tumors [37], is significantly down-regulated in smokers with expression correlating to that of mir-218 [36]. A second copy of miR-218 (miR-218-2) occurs within the SLIT3 locus located at 5q35.1, and expression of SLIT3 has also been shown to be down-regulated in lung cancer [38]. In general, chromosomal aberrations of chromosome 4 and 5 at multiple sites are frequent in lung cancer [39], [40]. Interestingly, miR-218-1 is located within FRA4D (aphidicolin type, common) and miR-218-2 is located within FRA5G (folic acid type, rare). Alteration of mir-218 levels diminishes the induction of the predicted mir-218 target MAFG in response to cigarette smoke condensate (CSC). miRNAs implicated in carcinogenesis are differentially expressed in the airway epithelium of smokers. This suggests that airway miRNA expression could potentially serve as an indicator of smoking-induced disease processes. Down-regulation of miRNAs in smokers could be related to the development of tobacco-related cancers [36].

Alcoholics also reveal a significantly higher frequency of fragile sites and chromosomal aberrations and the most frequent exchange types are deletions and polymorphic variations [41]. The specific chromosomal regions 1p36 (FRA1A), 1q21 (FRA1F), 2q21 (FRA2F), 2q31 (FRA2G), 5q31 (FRA5C), 7q22 (FRA7F), 7q32 (FRA7H) and 12q13 (FRA12A) are associated with chromosomal aberrations in alcoholics and many miRNAs, such as those in the mir-106b-25 cluster, are located within these fragile sites. Recently, it has been observed that the mir-106b-25 cluster (which includes miR-106b, miR-93 and miR-25, and is located at FRA7F) is over-expressed in hepatocellular carcinoma [42], a malignant tumor which is often related to high alcohol consumption [43]. In tumors with high expression of this cluster, a reduced expression of Bim, a miR-25 target and a critical regulator of apoptosis that plays an essential role in mammalian development, is observed [42]. This data suggests a possible correlation between alcohol assumption, chromosomal aberration of the region 7q22 and the mir-106b-25 cluster expression.

Since the prediction of miRNA targets still remains a challenge [44], the reported data may help to highlight significant correlations between the miRNAs and their predicted targets and to elucidate the role of miRNAs in cancer and other diseases, due to their genomic locations.

For example, rearrangements of chromosome 5, especially 5p gain, are often related to cervical cancer [45], and a miRNA located at 5p13.3, miR-579, is predicted to target the genes PTGS2 and IRF1, which are involved in cervical cancer. Moreover this miRNA is co-localized with RNASEN (Drosha), a gene which plays a key role in miRNA biogenesis and whose over-expression is observed in cervical cancer [45]. These data are consistent with the inactivation of IRF1 in various cancers.

Chromosome 19 has the highest gene density of all human chromosomes, more than double the genome-wide average, and the greatest incidence of miRNAs in fragile regions. The high number of miRNAs could be due to the high number of repeats which also may contribute to chromosome fragility (as we previously discussed). A telomeric association of the long arms of chromosome 19 (19q13.4) has been associated to Premature Ovarian Failure (POF) [46]. Our analysis shows a possible involvement of several members of the miR-515 family (such as miR-515-5p, miR-519 and miR-520a-3p/b/c-3p/d-3p/e/g/h), located at 19q13.4, in POF, since they are predicted to target FMR1 and FOXL2, two genes which are associated to POF [47], [48].

In conclusion, the presented data underline the importance of having detailed information about the miRNAs co-localization with genome fragile sites and unstable regions, in order to formulate hypoteses of targeting and involvement of miRNAs in diseases and biological processes. However, our results also reveal that fragile regions appear to be also denser in protein coding genes. It is when chromosome effects and interactions are allowed that significant differences in distribution between miRNAs and genes are observed. This demonstrates that the mechanisms regulating these unstable regions are more complex than what an analysis pooling all sites together could reveal. A local analysis of the incidence of miRNAs and genes is required.

All the collected data are available as supplementary material, and are being integrated into miRò, a web-based environment which provides users with powerful query tools for finding non-trivial associations among heterogeneous data such as miRNAs, processes, functions, diseases and expression profiles [49]. Such integration will make easier the study of the relationships between miRNAs and unstable regions, by allowing cross-checks between data sets and the extraction of non-trivial associations by the use of data mining facilities.

As a final note we emphasize that most fragile sites boundaries have not been characterized yet and are not known. As a result, in our study and following previous research [4], [50], [51], [52], [53], we mapped fragile sites to chromosomal bands and divided the 23 chromosomes into fragile and non-fragile regions (see Materials and Methods for further details). Therefore, some of the miRNAs mapping close to a fragile site may be in reality megabase pairs away. However, it is not known how close to a fragile site a miRNA must be in order for it to be affected. This limitation of our study demonstrates the need for further work in this area and for a more specific mapping of fragile sites. Despite these limitations, we feel that the approach we took to build the dataset is the best that can be applied at the moment.

## Materials and Methods

### The datasets

The set of the human miRNA genes is based on the release 13.0 (March 2009) of miRBase [54] and contains 715 miRNAs.

The fragile sites set consists of 118 genomic regions retrieved from NCBI Gene repository based on the Build 36.3 of the human genome. Most fragile sites boundaries have not been characterized yet. Data are available for only few of them, but this data is often susceptible of changes. For example, the fragile region FRA3B had been characterized as a large region of genomic instability covering 500 kb [55], [56], [57], [58]. However, Becker et al. [59], report that fragility at FRA3B actually extends over a 4 Mb region containing five genes.

For this reason, consistent with previous work [4], [50], [51], [52], [53] and with the NCBI MapViewer data, we mapped fragile sites to specific chromosomal bands. We then divided the 23 chromosomes into regions by looking at their sequential positioning (e.g., two *sequential* bands associated with fragile sites are grouped together to form a fragile region, and so on). We then determined the size of each region and classified the regions as having or not a fragile site by creating a fragile dummy variable (if a region has at least one fragile site the dummy will take the value 1, and if the region does not contain any fragile site the dummy takes the value 0). This way we created the 205 fragile and non-fragile regions used in our analysis.

The translocation breakpoints related to cancer come from TICdb [60] and their location is obtained by Ensembl 54. Fragile sites, translocation breakpoints and miRNAs are also annotated with information on the diseases in which they are known or predicted to be involved. These data come from miR2Disease (experimentally supported miRNA-disease associations) [61], miRò (predicted miRNA-disease associations) [49], OMIM, Mitelman Database of Chromosome Aberrations in Cancer [62] and the Chromosomal Variation in Man website (a database which contains a review of the literature on all common and rare chromosomal alterations and abnormalities) [63]. MicroRNA precursors structurally derived from transposable elements are obtained from the microTranspoGene database [29]. Finally, the CpG islands and the repetitive sequences are retrieved from the NCBI database.

### Statistical Analysis

Due to the discrete nature of the data, we used Poisson models to test whether miRNAs and protein coding genes appear more frequently in fragile sites. In these separate analyses, “events” were defined as the number of miRNAs or as the number of protein coding genes in each genomic region (depending on whether we are studying miRNA or gene incidence). We control for the size of genomic regions by directly accounting for the differential exposure due to region length (the length of each region is known and regions can vary significantly in size). We have also accounted for chromosome differences and allowed each chromosome to contain differing densities of miRNA and genes, irrespective of site fragility.

We tested the use of chromosome independent and non-independent random effects, RE, and of chromosome specific fixed effects (FE). After comparing model statistics (Bayesian Information Criterion, BIC, and Akaike Information Criterion, AIC), and after performing the Hausman test (5% significance), we conclude that chromosome dummies provided a better representation of the data for both miRNA and genes. We note however that the use of random effects does not alter significantly the results both in terms of parameter values and in terms of inference (when one effect is significant in one formulation it is also significant under the alternative formulation, and vice-versa; see Tables S6, S7, and S8 for an example comparing results from RE and FE version of the same model). Differences in inference could be present as RE models can at times be more efficient and have lower standard errors. We did not observe any significant difference also in inference (Tables S6, S7, and S8).

We note also that FE models are always consistent and that these are statistically a very reasonable formulation in our context because of the reduced number of cross-sectional units (23 chromosomes). As a result, the FE formulation does not require the use of an excessive number of parameters that could “consume” degrees of freedom during estimation and inference. At the same time it avoids any distributional assumptions on chromosome effects and it does not require that the random effects and model error terms be orthogonal. Finally, with a FE formulation we were able to flexibly include or exclude specific effects depending on their statistical significance, a task that is extremely difficult with RE (with RE once the chromosome effect is included all chromosomes have a differing parameter, even when not statistically significantly).

Hence, the results reported are for the models including chromosome dummies.

We further tested whether a *Zero-Inflated Poisson* (ZIP) model better fits the miRNA data (more than 30% of the miRNA observations are zero; protein coding genes do not present this characteristic and do not pose a problem for the use of standard Poisson models). Comparing the fit (BIC and AIC) of a ZIP model against that of traditional Poisson we concluded that the ZIP model is preferred (the ZIP model considers a Logit formulation for the zero-inflation component, with a constant and length as inflation variables; it separately models the zero case in the data and is especially useful in obtaining better parameter estimates when the data presents an excessive number of zeros, a feature standard Poisson models cannot easily accommodate).

Because of these results, the parameters reported for the miRNA variable are from the estimation of the ZIP model (for genes, the standard Poisson performed the best, as expected). We also analyzed for overdispersion of miRNA and genes data and concluded it was not a significant problem making Poisson (or ZIP) models adequate for modeling miRNA and genes.

In a separate analysis, to test whether fragile sites are associated with a higher incidence of repeats and CpG islands we estimated a Logit model of site fragility using repeats and CpG islands as predictors. The “event” to be modeled in this analysis is whether a genomic region is classified as fragile or not (a yes/no type of variable for which the Logit is the adequate model; we no longer have a count variable). We also tested for the use of RE or FE (chromosome dummies) to account for heterogeneity in incidence of fragile sites across chromosomes, and we found that chromosome dummies provided the best fit (however no significant difference in the main coefficients and inference between the two alternatives was found; in addition, the considerations regarding the RE and FE formulations we presented before also applied to this case).

Conversely, we have modeled the number of repeats and the number of CpG islands as a function of site fragility using standard Poisson models (no excessive number of zeros or overdispersion observed; indeed a ZIP model did not fit the data better than the standard Poisson model by comparing AIC and BIC fit measures). The analyses of site fragility and of CpG and Repeats are complementary as there is not clear causality relationship between these variables (i.e., it is unclear whether site fragility is caused by the presence of higher number of repeats and CpG islands or whether the reverse is true).

All computations were completed using STATA v10.0. We report the incidence rate ratio (IRR) and the two-sided 95% confidence intervals of the IRR. An IRR significantly >1 indicates an increase in the number of the dependent variable (either miRNA or genes, depending on the analysis) within a region of a certain type (e.g., in the miRNA model a “Fragile IRR” >1 means that fragile regions are more likely to contain miRNA than non-fragile sites).

We note that the statistical inference performed considered always a 5% significance level. In addition, standard errors were computed using the Observed Information Matrix method for most parameters. When estimating the RE formulation standard errors of the random effects (either for the intercept and for the “slope” parameters, as is the case of the parameter associated with the fragile dummy) the random effects and corresponding standard errors were estimated using the Best Linear Unbiased Predictor (BLUP) and the ‘*predict*’ function in STATA associated with the ‘*xtmepoisson*’ or ‘*xtmelogit*’ fucntions.

When performing statistical testing on parameters, we used a t-test with (*N*–*k*) degrees of freedom (where *N* is the number of observations and *k* the number of parameters of the estimated model). When testing differences of parameters we also used a t-test and estimated the standard error of the difference using the delta method (if coming from two different samples assumed that samples were independent).

## Supporting Information

Table S1 Summarizing table.
(0.08 MB DOC)

Table S2 Descriptive Statistics by Chromosome.
(0.06 MB DOC)

Table S3 Model Results: Chromosome-Specific Fragile Incidence Rate Ratios (IRR). ZIP model for miRNA and standard Poisson model for genes; model controls for differential exposure due to length and chromosome heterogeneity. *Chromosomes not listed showed no significant interactions with the fragile variable for both the miRNA and genes models (no significant differences in the missing chromosomes for fragile vs. non-fragile regions; in such case interactions have been removed from the final model formulation); absent values for a chromosome listed the table means the results were not significant for the specific model in which the value is absent (i.e., either for the miRNA model or for the genes model) and that the interaction has been removed from the final model formulation. For the miRNA we report conditional IRRs (unconditional IRRs available from the authors upon request).
(0.04 MB DOC)

Table S4 Model Results: Chromosome-Specific Fragile IRR when Including Repeats and CpGs. ZIP model for miRNA and standard Poisson model for genes; model controls for differential exposure due to length and chromosome heterogeneity. *Chromosomes not listed showed no significant interactions with the fragile variable for both the miRNA and genes models (no significant differences in the missing chromosomes for fragile vs. non-fragile regions; in such case interactions have been removed from the final model formulation); absent values for a chromosome listed the table means the results were not significant for the specific model in which the value is absent (i.e., either for the miRNA model or for the genes model) and that the interaction has been removed from the final model formulation. For the miRNA we report conditional IRRs (unconditional IRRs available from the authors upon request).
(0.04 MB DOC)

Table S5 Chromosome IRR Results for the Baseline Incidence of miRNA and Genes. We report on the ZIP model for miRNA and the Poisson model for genes; both models include chromosome dummies and the statistically significant interactions between chromosomes and site fragility, repeats, and CpG island effects; models also control for the differential exposure associated to differing site lengths. *These are Chromosome IRRs compared to the overall mean incidence of miRNAs or Genes (respectively) across all chromosomes; for example, the value of IRRmiRNA  = 3.551 for chromosome 14 means that in this chromosome there are about 3.6 times more miRNAs than the average miRNA across all chromosomes (irrespective if it is a fragile site or not and after accounting for the effect of fragile sites, Repeats and CpGs).
(0.06 MB DOC)

Table S6 Comparing the Fixed and Random Effects results. Estimates and Inference of Chromosome-Specific effects for the Model on Genes (Poisson model controlling for length and for site fragility). *Effects in bold are statistically significant at 5% significance level.
(0.09 MB DOC)

Table S7 Comparing the Fixed and Random Effects results. Estimates and Inference of Fragile Dummy Variable for the Models on miRNA and Genes (Poisson models controlling for length and for site fragility).
(0.04 MB DOC)

Table S8 Comparing the Fixed and Random Effects results. Estimates and Inference of Chromosome-Specific effects for the Model on miRNA (Poisson model controlling for length and for site fragility). *Effects in bold are statistically significant at 5% significance level.
(0.09 MB DOC)

## References

[1] Lukusa T, Fryns JP (2008). Human chromosome fragility.. Biochimica et Biophysica Acta.

[2] Durkin SG, Glover TW (2007). Chromosome fragile sites.. Annu Rev Genet.

[3] Glover TW (2006). Common fragile sites.. Cancer Letters.

[4] Burrow AA, Williams LE, Pierce LC, Wang YH (2009). Over half of breakpoints in gene pairs involved in cancer-specific recurrent translocations are mapped to human chromosomal fragile sites.. BMC Genomics.

[5] Huppi K, Volfovsky N, Mackiewicz M, Runfola T, Jones TL (2007). MicroRNAs and genomic instability.. Semin Cancer Biol.

[6] Calin GA, Sevignani C, Dumitru CD, Hyslop T, Noch E (2004). Human microRNA genes are frequently located at fragile sites and genomic regions involved in cancers.. Proc Natl Acad Sci USA.

[7] Bartel DP (2004). MicroRNAs: genomics, biogenesis, mechanism, and function.. Cell.

[8] Lytle JR, Yario TA, Steitz JA (2007). Target mRNAs are repressed as efficiently by microRNA-binding sites in the 5′ UTR as in the 3′ UTR.. Proc Natl Acad Sci USA.

[9] Forman JJ, Legesse-Miller A, Coller HA (2008). A search for conserved sequences in coding regions reveals that the let-7 microRNA targets Dicer within its coding sequence.. Proc Natl Acad Sci USA.

[10] Kloosterman WP, Plasterk RH (2006). The diverse functions of microRNAs in animal development and disease.. Dev Cell.

[11] Bushati N, Cohen SM (2007). MicroRNA functions.. Annu Rev Cell Dev Biol.

[12] Gangaraju VK, Lin H. MicroRNAs: key regulators of stem cells.. Nat Rev Mol Cell Biol.

[13] Lu J, Getz G, Miska EA, Alvarez-Saavedra E, Lamb J (2005). MicroRNA expression profiles classify human cancers.. Nature.

[14] Calin GA, Dumitru CD, Shimizu M, Bichi R, Zupo S (2002). Frequent deletions and down-regulation of micro-RNA genes miR15 and miR16 at 13q14 in chronic lymphocytic leukemia.. Proc Natl Acad Sci USA.

[15] Cimmino A, Calin GA, Fabbri M, Iorio MV, Ferracin M (2005). miR-15 and miR-16 induce apoptosis by targeting BCL2.. Proc Natl Acad Sci USA.

[16] Brueckner B, Stresemann C, Kuner R, Mund C, Musch T (2007). The human let-7a-3 locus contains an epigenetically regulated microRNA gene with oncogenic function.. Cancer Res.

[17] Bednarek AK, Keck-Waggoner CL, Daniel RL, Laflin KJ, Bergsagel PL (2001). WWOX, the FRA16D gene, behaves as a suppressor of tumor growth.. Cancer Res.

[18] Lewandowska U, Zelazowski M, Seta K, Byczewska M, Pluciennik E (2009). WWOX, the tumour suppressor gene affected in multiple cancers.. Physiol Pharmacol.

[19] Sutherland GR (2003). Rare fragile sites.. Cytogenet Genome Res.

[20] Sutherland GR, Richards RI (1995). The molecular basis of fragile sites in human chromosomes.. Curr Opin Genet Dev.

[21] Yu S, Mangelsdorf M, Hewett D, Hobson L, Baker E (1997). Human chromosomal fragile site FRA16B is an amplified AT-rich minisatellite repeat.. Cell.

[22] Hewett DR, Handt O, Hobson L, Mangelsdorf M, Eyre HJ (1998). FRA10B structure reveals common elements in repeat expansion and chromosomal fragile site genesis.. Mol Cell.

[23] Morelli C, Karayianni E, Magnanini C, Mungall AJ, Thorland E (2002). Cloning and characterization of the common fragile site FRA6F harboring a replicative senescence gene and frequently deleted in human tumors.. Oncogene.

[24] Smit AF (1996). The origin of interspersed repeats in the human genome.. Curr Opin Genet Dev.

[25] Smalheiser NR, Torvik VI (2005). Mammalian microRNAs derived from genomic repeats.. Trends Genet.

[26] Smalheiser NR, Torvik VI (2006). Alu elements within human mRNAs are probable microRNA targets.. Trends Genet.

[27] Sevignani C, Calin GA, Nnadi SC, Shimizu M, Davuluri RV (2007). MicroRNA genes are frequently located near mouse cancer susceptibility loci.. Proc Natl Acad Sci U S A.

[28] Ferro A, Giugno R, Laganà A, Pulvirenti A, Russo F (2009). Mapping miRNA genes on human fragile sites and translocation breakpoints..

[29] Levy A, Sela N, Ast G (2008). TranspoGene and microTranspoGene: transposed elements influence on the transcriptome of seven vertebrates and invertebrates.. Nucleic Acids Res.

[30] Göransson M, Andersson MK, Forni C, Ståhlberg A, Andersson C (2009). The myxoid liposarcoma FUS-DDIT3 fusion oncoprotein deregulates NF-kappaB target genes by interaction with NFKBIZ.. Oncogene.

[31] Dahéron L, Veinstein A, Brizard F, Drabkin H, Lacotte L (2001). Human LPP gene is fused to MLL in a secondary acute leukemia with a t(3;11) (q28;q23).. Genes Chromosomes Cancer.

[32] Borchert GM, Lanier W, Davidson BL (2006). RNA polymerase III transcribes human microRNAs.. Nat Struct Mol Biol.

[33] Grover D, Mukerji M, Bhatnagar P, Kannan K, Brahmachari SK (2004). Alu repeat analysis in the complete human genome: trends and variations with respect to genomic composition.. Bioinformatics.

[34] Lehnert S, Van Loo P, Thilakarathne PJ, Marynen P, Verbeke G (2009). Evidence for Co-Evolution between Human MicroRNAs and Alu-Repeats.. PLoS One.

[35] Lujambio A, Calin GA, Villanueva A, Ropero S, Sánchez-Céspedes M (2008). A microRNA DNA methylation signature for human cancer metastasis.. Proc Natl Acad Sci USA.

[36] Schembria F, Sridhar S, Perdomo C, Gustafson AM, Zhang X (2009). MicroRNAs as modulators of smoking-induced gene expression changes in human airway epithelium.. Proc Natl Acad Sci USA.

[37] Dallol A, Da Silva NF, Viacava P, Minna JD, Bieche I (2002). SLIT2, a human homologue of the Drosophila Slit2 gene, has tumor suppressor activity and is frequently inactivated in lung and breast cancers.. Cancer Res.

[38] Yanaihara N, Caplen N, Bowman E, Seike M, Kumamoto K (2006). Unique microRNA molecular profiles in lung cancer diagnosis and prognosis.. Cancer Cell.

[39] Shivapurkar N, Virmani AK, Wistuba II, Milchgrub S, Mackay B (1999). Deletions of chromosome 4 at multiple sites are frequent in malignant mesothelioma and small cell lung carcinoma.. Clin Cancer Res.

[40] Mendes-da-Silva P, Moreira A, Duro-da-Costa J, Matias D, Monteiro C (2000). Frequent loss of heterozygosity on chromosome 5 in non-small cell lung carcinoma.. Mol Pathol.

[41] Demirhan O, Tastemir D (2008). Cytogenetic effects of ethanol on chronic alcohol users.. Alcohol Alcohol.

[42] Li Y, Tan W, Neo TW, Aung MO, Wasser S Role of the miR-106b-25 microRNA cluster in hepatocellular carcinoma.. Cancer Sci.

[43] Schütte K, Bornschein J, Malfertheiner P (2009). Hepatocellular carcinoma: epidemiological trends and risk factors.. Dig Dis.

[44] Bartel DP (2009). MicroRNAs: target recognition and regulatory functions.. Cell.

[45] Scotto L, Narayan G, Nandula SV, Subramaniyam S, Kaufmann AM (2008). Integrative genomics analysis of chromosome 5p gain in cervical cancer reveals target over-expressed genes, including Drosha.. Mol Cancer.

[46] Zahed L, Darwiche N, Batanian JR, Awwad J (2002). Homologous telomere association of 19q in a female with premature ovarian failure.. Clin Genet.

[47] Vujovic S (2009). Aetiology of premature ovarian failure.. Menopause Int.

[48] Laissue P, Lakhal B, Benayoun BA, Dipietromaria A, Braham R (2009). Functional evidence implicating FOXL2 in non-syndromic premature ovarian failure and in the regulation of the transcription factor OSR2.. J Med Genet.

[49] Laganà A, Forte S, Giudice A, Arena MR, Puglisi PL (2009). miRò: a miRNA knowledge base..

[50] Popescu NC, Zimonjic D, DiPaolo JA (1990). Viral integration, fragile sites, and proto-oncogenes in human neoplasia.. Hum Genet.

[51] Matuszek G, Talebizadeh Z (2009). Autism genetic database (AGD): a comprehensive database including autism susceptibility gene-CNVs integrated with known noncoding RNAs and fragile sites.. BMC Medical Genetics 2009,.

[52] Ruiz-Herrera A, Castresana J, Robinson TJ (2006). Is mammalian chromosomal evolution driven by regions of genome fragility?. Genome Biology.

[53] Kim S, Kim N, Dong B, Boren D, Lee SA (2008). Integration Site Preference of Xenotropic Murine Leukemia Virus-Related Virus, a New Human Retrovirus Associated with Prostate Cancer.. Journal of Virology.

[54] Griffiths-Jones S, Saini HK, van Dongen S, Enright AJ (2008). miRBase: tools for microRNA genomics.. Nucleic Acids Res.

[55] Paradee W, Wilke CM, Wang L, Shridhar R, Mullins CM (1996). A 350-kb cosmid contig in 3p14.2 that crosses the t(3;8) hereditary renal cell carcinoma translocation breakpoint and 17 aphidicolin-induced FRA3B breakpoints.. Genomics.

[56] Rassool FV, Le Beau MM, Shen ML, Neilly ME, Espinosa R (1996). Direct cloning of DNA sequences from the common fragile site region at chromosome band 3p14.2.. Genomics.

[57] Wilke CM, Hall BK, Hoge A, Paradee W, Smith DI (1996). FRA3B extends over a broad region and contains a spontaneous HPV16 integration site: direct evidence for the coincidence of viral integration sites and fragile sites.. Hum Mol Genet.

[58] Zimonjic DB, Druck T, Ohta M, Kastury K, Croce CM (1997). Positions of chromosome 3p14.2 fragile sites (FRA3B) within the FHIT gene.. Cancer Res.

[59] Becker NA, Thorland EC, Denison SR, Phillips LA, Smith DI (2002). Evidence that instability within the FRA3B region extends four megabases.. Oncogene.

[60] Novo FJ, de Mendíbil IO, Vizmanos JL (2007). TICdb: a collection of gene-mapped translocation breakpoints in cancer.. BMC Genomics.

[61] Jiang Q, Wang Y, Hao Y, Juan L, Teng M (2009). miR2Disease: a manually curated database for microRNA deregulation in human disease.. Nucleic Acids Res.

[62] Mitelman F, Johansson B, Mertens F (2008). Mitelman Database of Chromosome Aberrations in Cancer.. http://cgap.nci.nih.gov/Chromosomes/Mitelman.

[63] Borgaonkar DS, Bolling DR, Partridge C, Ruddle FH, McKusick VA (1975). Chromosomal variation in man: catalog of chromosomal variants and anomalies.. Cytogenet Cell Genet.

