# Supplementary material for: Variability in the Incidence of miRNAs and Genes in Fragile Sites and the Role of Repeats and CpG Islands in the Distribution of Genetic Material
Source: PLoS One. 2010 Jun 17;5(6):e11166. doi: 10.1371/journal.pone.0011166 (PMC2887363; doi:10.1371/journal.pone.0011166)
Supplement: Table S1 — Summarizing table. (0.08 MB DOC) [file pone.0011166.s001.doc]

| **Chr** | **Frag. Sites (FS)** | **miRNAs** | **miRNAs in FS** | **Translocation Breakpoints (TB)** | **miRNAs in TB** | **miRNAs in TB and FS** | **Protein coding genes** | **Prot. Cod. genes in FS** | **Repeats** | **Repeats in FS** | **CpG islands** | **CpG islands in FS** | **Chr size (Mbp)** | **FS (%)** |
| --- | --- | --- | --- | --- | --- | --- | --- | --- | --- | --- | --- | --- | --- | --- |
| 1 | 13 | 50 | 22 | 25 | 15 | 3 | 2169 | 1130 | 355316 | 186093 | 26921 | 14955 | 247 | 54 |
| 2 | 11 | 26 | 8 | 16 | 10 | 5 | 1403 | 521 | 342442 | 90810 | 22351 | 6985 | 243 | 26,25 |
| 3 | 4 | 31 | 2 | 17 | 16 | 2 | 1134 | 129 | 280546 | 27642 | 15998 | 1308 | 200 | 11,9 |
| 4 | 4 | 27 | 5 | 13 | 7 | 0 | 911 | 174 | 264422 | 60309 | 14956 | 3491 | 191 | 20,57 |
| 5 | 7 | 31 | 14 | 12 | 13 | 10 | 883 | 228 | 254205 | 87322 | 15697 | 5867 | 181 | 33,2 |
| 6 | 7 | 17 | 3 | 21 | 8 | 1 | 1118 | 194 | 236750 | 45302 | 16245 | 2919 | 171 | 17,89 |
| 7 | 10 | 34 | 15 | 17 | 11 | 5 | 1125 | 602 | 231207 | 98478 | 19129 | 12233 | 159 | 38,74 |
| 8 | 5 | 31 | 8 | 14 | 13 | 1 | 843 | 234 | 291815 | 41039 | 18505 | 3507 | 146 | 17,8 |
| 9 | 6 | 27 | 6 | 17 | 10 | 2 | 891 | 105 | 184286 | 29117 | 14583 | 1722 | 140 | 19,14 |
| 10 | 8 | 24 | 7 | 16 | 12 | 4 | 885 | 386 | 198365 | 82845 | 14893 | 5115 | 135 | 40,88 |
| 11 | 9 | 25 | 7 | 23 | 12 | 6 | 1454 | 485 | 198719 | 56750 | 14310 | 5409 | 134 | 25,07 |
| 12 | 5 | 30 | 11 | 14 | 11 | 5 | 1150 | 466 | 205584 | 80618 | 14943 | 6653 | 132 | 34,69 |
| 13 | 4 | 14 | 2 | 5 | 2 | 0 | 375 | 52 | 136072 | 42640 | 8230 | 2010 | 114 | 24,91 |
| 14 | 2 | 62 | 2 | 13 | 59 | 1 | 707 | 91 | 131359 | 20178 | 9710 | 1177 | 106 | 12,73 |
| 15 | 1 | 25 | 3 | 8 | 10 | 0 | 755 | 75 | 123570 | 15475 | 9705 | 1065 | 100 | 9,5 |
| 16 | 5 | 15 | 6 | 12 | 11 | 6 | 1050 | 437 | 141592 | 28950 | 14286 | 2270 | 89 | 16,17 |
| 17 | 2 | 34 | 3 | 22 | 23 | 1 | 1310 | 40 | 133783 | 8806 | 16441 | 730 | 79 | 6,8 |
| 18 | 3 | 9 | 3 | 6 | 4 | 0 | 320 | 74 | 102863 | 24224 | 6267 | 1219 | 76 | 22,89 |
| 19 | 2 | 80 | 79 | 14 | 23 | 23 | 1545 | 1483 | 110559 | 89345 | 19417 | 17181 | 64 | 72,65 |
| 20 | 2 | 23 | 0 | 4 | 11 | 0 | 195 | 36 | 102514 | 9537 | 8599 | 492 | 62 | 10,16 |
| 21 | 0 | 5 | 0 | 3 | 1 | 0 | 280 | 0 | 49984 | 0 | 4838 | 0 | 47 | 0 |
| 22 | 2 | 16 | 7 | 9 | 12 | 4 | 530 | 287 | 60595 | 30107 | 8063 | 4556 | 50 | 32,8 |
| X | 6 | 79 | 29 | 9 | 23 | 8 | 912 | 217 | 210745 | 30747 | 13461 | 2125 | 155 | 14,5 |
| **TOT** | **118** | **715** | **242** | **310** | **317** | **87** | **21945** | **7446** | **4347293** | **1186334** | **327548** | **102989** |  |  |
