# Supplementary material for: Variability in the Incidence of miRNAs and Genes in Fragile Sites and the Role of Repeats and CpG Islands in the Distribution of Genetic Material
Source: PLoS One. 2010 Jun 17;5(6):e11166. doi: 10.1371/journal.pone.0011166 (PMC2887363; doi:10.1371/journal.pone.0011166)
Supplement: Table S2 — Descriptive Statistics by Chromosome. (0.06 MB DOC) [file pone.0011166.s002.doc]

| Chromosome | miRNA | | Protein Coding Genes | | Number  of Regions | Average Length of Each Region |
| --- | --- | --- | --- | --- | --- | --- |
|  | Mean | Standard Deviation | Mean | Standard Deviation |  |  |
| 1 | 2.78 | 3.39 | 120.50 | 117.54 | 18 | 13.73 |
| 2 | 1.24 | 1.87 | 66.81 | 57.84 | 21 | 11.57 |
| 3 | 3.44 | 3.57 | 126.00 | 145.93 | 9 | 22.09 |
| 4 | 3.38 | 4.50 | 113.88 | 110.44 | 8 | 23.93 |
| 5 | 3.10 | 2.96 | 88.30 | 85.08 | 10 | 18.14 |
| 6 | 1.13 | 1.96 | 74.53 | 120.95 | 15 | 11.40 |
| 7 | 1.79 | 2.27 | 59.21 | 52.30 | 19 | 8.36 |
| 8 | 3.88 | 4.16 | 105.38 | 179.56 | 8 | 18.35 |
| 9 | 3.00 | 3.35 | 99.00 | 99.51 | 9 | 15.59 |
| 10 | 1.85 | 2.03 | 68.08 | 50.09 | 13 | 10.43 |
| 11 | 1.92 | 2.60 | 111.85 | 119.30 | 13 | 10.35 |
| 12 | 5.00 | 1.90 | 191.67 | 109.99 | 6 | 22.12 |
| 13 | 1.75 | 2.49 | 46.88 | 45.91 | 8 | 14.63 |
| 14 | 15.50 | 27.05 | 176.75 | 153.25 | 4 | 26.63 |
| 15 | 8.33 | 4.62 | 251.67 | 153.05 | 3 | 33.50 |
| 16 | 1.67 | 1.94 | 116.67 | 111.11 | 9 | 9.80 |
| 17 | 6.80 | 5.97 | 262.00 | 278.15 | 5 | 15.76 |
| 18 | 1.50 | 1.87 | 53.33 | 47.09 | 6 | 12.68 |
| 19 | 26.67 | 30.92 | 515.00 | 408.96 | 3 | 21.27 |
| 20 | 4.60 | 8.71 | 39.00 | 40.88 | 5 | 12.44 |
| 21 | 5.00 | — | 280.00 | — | 1 | 47.00 |
| 22 | 4.00 | 4.69 | 132.50 | 86.82 | 4 | 12.43 |
| X | 9.88 | 11.56 | 114.00 | 150.30 | 8 | 19.20 |
