# Supplementary material for: Variability in the Incidence of miRNAs and Genes in Fragile Sites and the Role of Repeats and CpG Islands in the Distribution of Genetic Material
Source: PLoS One. 2010 Jun 17;5(6):e11166. doi: 10.1371/journal.pone.0011166 (PMC2887363; doi:10.1371/journal.pone.0011166)
Supplement: Table S3 — Model Results: Chromosome-Specific Fragile Incidence Rate Ratios (IRR). ZIP model for miRNA and standard Poisson model for genes; model controls for differential exposure due to length and chromosome heterogeneity. *Chromosomes not listed showed no significant interactions with the fragile variable for both the miRNA and genes models (no significant differences in the missing chromosomes for fragile vs. non-fragile regions; in such case interactions have been removed from the final model formulation); absent values for a chromosome listed the table means the results were not significant for the specific model in which the value is absent (i.e., either for the miRNA model or for the genes model) and that the interaction has been removed from the final model formulation. For the miRNA we report conditional IRRs (unconditional IRRs available from the authors upon request). (0.04 MB DOC) [file pone.0011166.s003.doc]

| Chromosome | miRNA | | Protein Coding Genes | |
| --- | --- | --- | --- | --- |
|  | Fragile IRR | 95% Confidence Interval | Fragile IRR | 95% Confidence Interval |
| 2 | — | — | 1.659 | [1.489, 1.849] |
| 5 | — | — | 0.703 | [0.604, 0.817] |
| 7 | — | — | 1.816 | [1.615, 2.042] |
| 8 | — | — | 1.785 | [1.535, 2.076] |
| 9 | — | — | 0.566 | [0.462, 0.694] |
| 11 | — | — | 1.503 | [1.348, 1.676] |
| 12 | — | — | 1.293 | [1.149, 1.454] |
| 13 | — | — | 0.441 | [0.329, 0.591] |
| 14 | 0.244 | [0.059, 1.000] | — | — |
| 16 | 6.560 | [2.123, 20.277] | 3.654 | [3.232, 4.131] |
| 17 | — | — | 0.428 | [0.313, 0.586] |
| 19 | 29.391 | [4.089, 211.248] | 8.899 | [6.902, 11.473] |
| 20 | — | — | 2.009 | [1.399, 2.885] |
| 22 | — | — | 2.398 | [2.021, 2.845] |
| X | 4.397 | [2.763, 6.995] | 1.810 | [1.554, 2.108] |
