# Supplementary material for: Variability in the Incidence of miRNAs and Genes in Fragile Sites and the Role of Repeats and CpG Islands in the Distribution of Genetic Material
Source: PLoS One. 2010 Jun 17;5(6):e11166. doi: 10.1371/journal.pone.0011166 (PMC2887363; doi:10.1371/journal.pone.0011166)
Supplement: Table S5 — Chromosome IRR Results for the Baseline Incidence of miRNA and Genes. We report on the ZIP model for miRNA and the Poisson model for genes; both models include chromosome dummies and the statistically significant interactions between chromosomes and site fragility, repeats, and CpG island effects; models also control for the differential exposure associated to differing site lengths. *These are Chromosome IRRs compared to the overall mean incidence of miRNAs or Genes (respectively) across all chromosomes; for example, the value of IRRmiRNA = 3.551 for chromosome 14 means that in this chromosome there are about 3.6 times more miRNAs than the average miRNA across all chromosomes (irrespective if it is a fragile site or not and after accounting for the effect of fragile sites, Repeats and CpGs). (0.06 MB DOC) [file pone.0011166.s005.doc]

| Chromosome | miRNA | | Protein Coding Genes | |
| --- | --- | --- | --- | --- |
|  | IRR* | 95% Confidence Interval | IRR* | 95% Confidence Interval |
| 1 | — | — | 1.215 | [1.158, 1.274] |
| 2 | — | — | 0.778 | [0.727, 0.834] |
| 3 | — | — | — | — |
| 4 | — | — | 0.896 | [0.829, 0.967] |
| 5 | — | — | 0.899 | [0.829, 0.976] |
| 6 | 0.599 | [0.362, 0.992] | — | — |
| 7 | — | — | 0.892 | [0.818, 0.973] |
| 8 | 1.812 | [1.144, 2.870] | 0.718 | [0.631, 0.818] |
| 9 | — | — | — | — |
| 10 | — | — | — | — |
| 11 | — | — | 1.515 | [1.419, 1.616] |
| 12 | — | — | 1.152 | [1.063, 1.248] |
| 13 | — | — | 0.583 | [0.522, 0.651] |
| 14 | 3.551 | [2.625, 4.803] | — | — |
| 15 | — | — | — | — |
| 16 | — | — | — | — |
| 17 | 1.717 | [1.127, 2.616] | 1.620 | [1.503, 1.745] |
| 18 | — | — | 0.660 | [0.591, 0.737] |
| 19 | — | — | 0.478 | [0.373, 0.614] |
| 20 | 1.986 | [1.213, 3.251] | 0.314 | [0.267, 0.370] |
| 21 | — | — | 0.673 | [0.595, 0.761] |
| 22 | — | — | — | — |
| X | 3.164 | [2.148, 4.661] | 0.870 | [0.793, 0.955] |
