# Supplementary material for: Variability in the Incidence of miRNAs and Genes in Fragile Sites and the Role of Repeats and CpG Islands in the Distribution of Genetic Material
Source: PLoS One. 2010 Jun 17;5(6):e11166. doi: 10.1371/journal.pone.0011166 (PMC2887363; doi:10.1371/journal.pone.0011166)
Supplement: Table S6 — Comparing the Fixed and Random Effects results. Estimates and Inference of Chromosome-Specific effects for the Model on Genes (Poisson model controlling for length and for site fragility). *Effects in bold are statistically significant at 5% significance level. (0.09 MB DOC) [file pone.0011166.s006.doc]

|  | Fixed Effects Model | | | Random Effects Model | | |
| --- | --- | --- | --- | --- | --- | --- |
| Chromosome | Parameter Estimate | [95% Conf. Interval] | | Parameter Estimate | [95% Conf. Interval] | |
|  |  | Lower Bound | Upper Bound |  | Lower Bound | Upper Bound |
| 1 | **0.135** | **0.089** | **0.181** | **0.137** | **-0.092** | **0.454** |
| 2 | **-0.200** | **-0.253** | **-0.147** | **-0.197** | **0.248** | **-0.542** |
| 3 | **-0.166** | **-0.224** | **-0.108** | **-0.163** | **0.219** | **-0.434** |
| 4 | **-0.375** | **-0.440** | **-0.310** | **-0.371** | **0.432** | **-1.051** |
| 5 | **-0.392** | **-0.459** | **-0.325** | **-0.388** | **0.451** | **-1.102** |
| 6 | -0.049 | -0.108 | 0.010 | -0.047 | 0.104 | -0.083 |
| 7 | -0.035 | -0.095 | 0.026 | -0.033 | 0.091 | -0.040 |
| 8 | **-0.178** | **-0.246** | **-0.110** | **-0.175** | **0.240** | **-0.460** |
| 9 | **-0.082** | **-0.148** | **-0.016** | **-0.080** | **0.143** | **-0.175** |
| 10 | **-0.122** | **-0.190** | **-0.055** | **-0.120** | **0.185** | **-0.295** |
| 11 | **0.431** | **0.379** | **0.483** | **0.432** | **-0.380** | **1.347** |
| 12 | **0.180** | **0.121** | **0.239** | **0.181** | **-0.123** | **0.602** |
| 13 | **-0.790** | **-0.892** | **-0.689** | **-0.778** | **0.868** | **-2.245** |
| 14 | -0.017 | -0.090 | 0.057 | -0.014 | 0.086 | 0.029 |
| 15 | **0.118** | **0.046** | **0.189** | **0.119** | **-0.049** | **0.428** |
| 16 | **0.555** | **0.495** | **0.616** | **0.555** | **-0.493** | **1.726** |
| 17 | **0.921** | **0.867** | **0.975** | **0.920** | **-0.864** | **2.814** |
| 18 | **-0.506** | **-0.616** | **-0.396** | **-0.497** | **0.597** | **-1.390** |
| 19 | **1.096** | **1.040** | **1.151** | **1.094** | **-1.037** | **3.339** |
| 20 | **-0.758** | **-0.899** | **-0.618** | **-0.738** | **0.857** | **-2.093** |
| 21 | -0.082 | -0.199 | 0.035 | -0.078 | 0.192 | -0.121 |
| 22 | **0.392** | **0.306** | **0.478** | **0.390** | **-0.303** | **1.259** |
| X | **-0.135** | **-0.200** | **-0.070** | **-0.132** | **0.195** | **-0.334** |
