# Supplementary material for: Variability in the Incidence of miRNAs and Genes in Fragile Sites and the Role of Repeats and CpG Islands in the Distribution of Genetic Material
Source: PLoS One. 2010 Jun 17;5(6):e11166. doi: 10.1371/journal.pone.0011166 (PMC2887363; doi:10.1371/journal.pone.0011166)
Supplement: Table S7 — Comparing the Fixed and Random Effects results. Estimates and Inference of Fragile Dummy Variable for the Models on miRNA and Genes (Poisson models controlling for length and for site fragility). (0.04 MB DOC) [file pone.0011166.s007.doc]

|  | Fixed Effects Model | | | Random Effects Model | | |
| --- | --- | --- | --- | --- | --- | --- |
|  | Parameter Estimate | [95% Conf. Interval] | | Parameter Estimate | [95% Conf. Interval] | |
|  |  | Lower Bound | Upper Bound |  | Lower Bound | Upper Bound |
| Fragile Effect (gene) | 0.297 | 0.266 | 0.328 | 0.298 | 0.267 | 0.328 |
| Fragile Effect (miRNA) | 0.383 | 0.228 | 0.538 | 0.312 | 0.141 | 0.483 |
