# Supplementary material for: Variability in the Incidence of miRNAs and Genes in Fragile Sites and the Role of Repeats and CpG Islands in the Distribution of Genetic Material
Source: PLoS One. 2010 Jun 17;5(6):e11166. doi: 10.1371/journal.pone.0011166 (PMC2887363; doi:10.1371/journal.pone.0011166)
Supplement: Table S8 — Comparing the Fixed and Random Effects results. Estimates and Inference of Chromosome-Specific effects for the Model on miRNA (Poisson model controlling for length and for site fragility). *Effects in bold are statistically significant at 5% significance level. (0.09 MB DOC) [file pone.0011166.s008.doc]

|  | Fixed Effects Model | | | Random Effects Model | | |
| --- | --- | --- | --- | --- | --- | --- |
| Chromosome | Parameter Estimate | [95% Conf. Interval] | | Parameter Estimate | [95% Conf. Interval] | |
|  |  | Lower Bound | Upper Bound |  | Lower Bound | Upper Bound |
| 1 | -0.216 | -0.512 | 0.081 | -0.168 | -0.442 | 0.105 |
| 2 | **-0.768** | **-1.156** | **-0.379** | **-0.656** | **-1.015** | **-0.296** |
| 3 | -0.344 | -0.697 | 0.009 | -0.276 | -0.615 | 0.063 |
| 4 | **-0.472** | **-0.852** | **-0.093** | **-0.390** | **-0.748** | **-0.031** |
| 5 | -0.321 | -0.680 | 0.038 | -0.258 | -0.597 | 0.082 |
| 6 | **-0.814** | **-1.291** | **-0.337** | **-0.663** | **-1.091** | **-0.236** |
| 7 | -0.114 | -0.459 | 0.232 | -0.069 | -0.397 | 0.259 |
| 8 | -0.060 | -0.414 | 0.295 | -0.017 | -0.360 | 0.326 |
| 9 | -0.157 | -0.537 | 0.222 | -0.105 | -0.468 | 0.259 |
| 10 | -0.310 | -0.719 | 0.099 | -0.242 | -0.622 | 0.138 |
| 11 | -0.211 | -0.607 | 0.185 | -0.153 | -0.528 | 0.222 |
| 12 | -0.046 | -0.411 | 0.319 | -0.006 | -0.354 | 0.342 |
| 13 | **-0.657** | **-1.184** | **-0.130** | **-0.514** | **-0.979** | **-0.049** |
| 14 | **0.971** | **0.721** | **1.222** | **0.966** | **0.712** | **1.220** |
| 15 | 0.132 | -0.261 | 0.525 | 0.155 | -0.226 | 0.537 |
| 16 | -0.271 | -0.779 | 0.236 | -0.193 | -0.658 | 0.272 |
| 17 | **0.692** | **0.355** | **1.028** | **0.674** | **0.335** | **1.012** |
| 18 | -0.656 | -1.312 | -0.001 | -0.472 | -1.019 | 0.076 |
| 19 | **1.553** | **1.295** | **1.811** | **1.531** | **1.305** | **1.757** |
| 20 | **0.526** | **0.117** | **0.935** | **0.501** | **0.097** | **0.906** |
| 21 | -0.685 | -1.561 | 0.192 | -0.420 | -1.079 | 0.240 |
| 22 | 0.312 | -0.182 | 0.807 | 0.296 | -0.175 | 0.766 |
| X | **0.841** | **0.618** | **1.064** | **0.850** | **0.625** | **1.074** |
